# Health Care Professionals’ Experiences in Telerehabilitation: Qualitative Content Analysis

**DOI:** 10.2196/40690

**Published:** 2023-04-19

**Authors:** Maria Qvarfordt, Evalill Nilsson, Lina Nilsson

**Affiliations:** 1 eHealth Institute Department of Medicine and Optometry, Faculty of Health and Life Sciences Linnaeus University Kalmar Sweden

**Keywords:** digitalization, eHealth, habilitation, health care digital encounters, telemedicine, telerehabilitation, HCP, health care professionals, experience, workflow, health care, accessibility, health care employees, perspectives, acceptability

## Abstract

**Background:**

The use of digital communication in Swedish health care has increased in an effort to make health care more accessible. At the organizational level, trust in digitalization has stabilized, but a certain degree of skepticism regarding technology appears to exist among health care employees.

**Objective:**

This study aimed to explore health care professionals’ (HCPs) experiences of digital communication with patients and colleagues in a habilitation context.

**Methods:**

Qualitative content analysis was used to analyze data derived from individual interviews.

**Results:**

The results revealed that there were mixed feelings regarding the digital format used at the habilitation center. Although some skepticism remained regarding the digital format, there seemed to be a parallel understanding of the motives and benefits of digitalization. Hence, positive aspects, such as increased health care accessibility, were identified. However, emphasis was placed on the considerations required to make digital consultations appropriate for each patient.

**Conclusions:**

Managing a workday influenced by the balance between digital and physical demands forces HCPs to adjust to the digital format and new ways of working. This requires HCPs to consider whether digital means are appropriate for communication in individual patient-specific cases.

## Introduction

Within health care, “habilitation” pertains to helping individuals with congenital developmental disabilities achieve or improve skills and functions necessary for daily living [1,2], whereas “rehabilitation” pertains to helping individuals regain skills, abilities, or knowledge that may have been lost or compromised because of illness or injury or after acquiring a disability [3]. Habilitation services include evaluations, assessments, monitoring, supervision, education, consultation, and coaching. These services can be provided in a physical setting at the habilitation clinic (HC) but also remotely through the use of digital tools (telemedicine) [4]. The latter is part of the ongoing health care digital transformation [5], where digital tools are increasingly used for patient diagnostics, monitoring, and treatment [6], for example, digital consultations [7]. In digital consultations, video can be used as a medium for communicating synchronously [8], but asynchronous communication may occur as well, for example, via text messages [9] and web portals [10].

Although telerehabilitation is an accepted telemedicine subfield [11], telehabilitation is not commonly used [4]. Rather, it seems to be considered a part of telerehabilitation, although the purpose and requirements may differ. Telerehabilitation has been shown to reduce health care and patient costs [12] and may have the potential to reduce patients’ time [12,13]. Adding to the benefits of digitally delivered health care, telerehabilitation has also been proposed to increase accessibility [12]. Despite these advantages, challenges remain, such as cases in which digital patient consultations are appropriate or sufficient [14] and addressing prevailing skepticism toward digital tools among health care professionals (HCPs) [12].

There is a lack of evidence on the impact and efficacy of digital tool use in practice, such as for the delivery of higher-quality health care [14,15]. For example, more evidence is needed on how digital communication in health care is best implemented as a replacement for “traditional” patient consultations and on how to run digital consultations most effectively. Is there a limit to when digital consultations would no longer mean an improvement in care but rather a deterioration of care [16]? As an increasingly digitalized working environment offers opportunities to reshape the traditional ways of providing health care [16], possible benefits and disadvantages for patients and HCPs merit further exploration [16], for example, addressing how digital tool use may influence HCPs’ working conditions [14]. Additional questions are raised regarding accessibility, as accessibility in telerehabilitation is associated with how digital tools are designed [17], for instance, with respect to patient inclusivity [18], which is one of the core concerns of habilitation services [3]. Thus, the objective of this study was to explore HCPs’ experiences of digital communication with patients and colleagues in a habilitation context.

## Methods

### Overview

Qualitative interviews were conducted to explore HCPs’ experiences at an HC in southern Sweden. The interviews were conducted using a data management process modified by Halcomb and Davidson [19]. The following report was inspired by the COREQ (Consolidated Criteria for Reporting Qualitative Studies) guidelines [20].

### Study Context

For the purposes of this study, individuals who are in contact with the HC for health care purposes are referred to as “patients.” For digital communication between patients and HCPs, but also between HCPs and other actors, such as other HCPs or other professions at different work sites, schools, or with close contacts—that is, parties involved in a patients’ everyday lives as part of the clinical setting—the term “video consultation” will be used. For digital communication between employees at the HC in organizational settings, such as when attending staff meetings or conferences, the term “videoconferencing” [21] will be used. In both instances, digital communication may be synchronous or asynchronous.

As part of the decentralized Swedish health care system, HCs are organized under the medical or social departments at a regional level [22]. HC employees work in multiprofessional teams that include, for example, nurses, physicians, physiotherapists, psychologists, and speech therapists. Although face-to-face consultations at the clinic were common practice before the COVID-19 pandemic, digital meetings also occurred to some extent, as this particular clinic had implemented video consultations between HCPs and patients before 2020. However, during the study period, all meetings, conferences, and patient visits at this HC were redirected and held via digital formats because of the restrictions imposed by the COVID-19 pandemic.

### Sampling Procedure and Participants

The HC in this study comprised several units, 3 of which were eligible for our study. The 2 ineligible units included the unit for children and youth (aged ≤18 years) and the Assistive Technology Center. The inclusion criteria for this study were employment at the HC and contact with patients (aged ≥18 years) and colleagues as part of the participants’ professional workday routine, as described in the *Study Context* section. The sampling procedure in this study followed a stratified purposeful process [23], as researchers aimed to assemble as heterogeneous a group as possible, with respect to participant profession and years active in the profession.

In September 2020, one researcher (MQ) contacted the operations developer of the HC to inform them about the study. The operations developer became our contact person during the study period and contacted the HC management to inform them about the study. When the operations developer received approval from the management that the study could proceed, HC employees were informed about the study via a presentation at a web-based workplace meeting by researchers (LN, EN, and MQ) in October 2020. If interested in participating, employees were requested to correspond with the operations developer, who forwarded potential participants’ contact information to 1 of the researchers (MQ). MQ contacted potential participants via email to provide written information about the study and to book interview appointments. The intention was to use a purposeful strategic sampling. A total of 11 participants signed up for the study, and further sampling was not necessary.

### Data Collection

By following an interview guide consisting of semistructured open-ended questions, individual interviews (N=11) were conducted in May and June 2021 by 2 researchers (MQ and LN) via a videoconference. During the interviews, 1 of the researchers (LN) took notes, and after every session, the researchers held a reflexive dialogue about what could be summarized from the interview in line with the analysis method [19].

The interview questions were arranged into 3 themes that were constructed to capture HCPs’ experiences of digital communication with colleagues and patients (Table 1).

**Table 1:** Interview questions arranged into 3 themes that concerned digital communication in habilitation.

Health care professionals’ experiences of digital communication in a habilitation context and theme	Examples of interview questions
Patient communication and digital patient communication (opening questions)	Describe in what ways you communicate with patients within your business today regarding patient notifications and follow-ups? What digital tools or facilities do you use to communicate digitally within the habilitation center?
Experiences with digital meetings and digital patient encounters	Keeping digital communication in mind: what opportunities do you believe are offered by the digital format? Keeping possible self-experienced challenges with digital communication in mind: how could the digital encounter with patients be improved?
Experiences of digital patient communication: making adjustments because of demands of the digital format	In what ways do you, as a health care professional, need to adjust your working day because of the use of digital tools such as digital meetings with patients? Describe whether there are any resources saved because of the use of digital communication tools? (eg, time, efficiency, and preparations)

Before the interviews, participants were informed orally of the study’s purpose in accordance with the informed consent document that was sent via email when scheduling the interview appointment. Only the audio from the interviews was recorded on an external audio-recording device. In addition to providing oral consent at the beginning of the recording, participants also emailed or mailed written informed consent to 1 of the researchers (MQ). Recordings were saved as audio files in a data-secure storage folder, which only the researchers had access to. Two of the interviews were not recorded because of failure of the recording device. However, note-taking from the interview sessions and memos from reflections were documented from these 2 interviews, just as they were for all other interviews.

### Data Analysis

The data management process, originally described by Halcomb and Davidson [19], is an iterative reflexive process that integrates the use of verbatim and nonverbatim transcription [19]. We used selective transcription [24], followed by qualitative content analysis, to analyze the data. Finally, a thematic review of the material enabled any necessary changes to be made to codes and themes and aimed for consensus among the researchers (MQ and LN). Codes emerged iteratively during the entire analysis process, and codes were added, deleted, or renamed as the process progressed. During the thematic review of the secondary content analysis, codes and themes were examined to determine the interactions and relationships between them and to ensure that they were a true representation of what had been expressed by respondents.

### Ethics Approval and Participation

All interviews were conducted on a voluntary basis, and participants could withdraw from the study at any stage, without being prompted to give an explanation as to why. The interviews were handled under the principle of confidentiality, and the transcripts were pseudonymized. All the participants provided oral and written consent to participate in the study. Professional backgrounds or participants’ specific work sites were not presented in the study to minimize the risk of participants being identified because of the small sample size in relation to the study context. This study was approved by the Swedish Ethical Review Authority (2021-01318).

## Results

A total of 11 participants (10 of whom were women) representing the most common professions at the HC participated in the study. Participants had worked for varying periods in their profession, ranging from 1 to 37 years. The interview length ranged between 36 and 56 minutes.

### Qualitative Analysis

Two main categories (*adapting to the digital working environment* and *the difference between replacement and complement*) and 4 subcategories (*cogwheels in the digitalization machinery: importance of support, knowledge, and preparation;* *workplace-accelerated digitalization: opportunities and challenges for professions*; *emphasizing the flexibility of digital communication: the digital format as a facilitator*; and *having case-by-case awareness: digital format suitability*) were identified, all emphasizing the overall theme of having to balance a digital-physical workday (Textbox 1).

**Main categories and subcategories relating to the main theme of balancing a digital-physical workday.**

- Adapting to the digital working environment
  Cogwheels in the digitalization machinery: importance of support, knowledge, and preparation
  Workplace-accelerated digitalization: opportunities and challenges for the professional role
- The difference between replacement and complement
  Emphasizing the flexibility of digital communication: the digital format as a facilitator
  Having case-by-case awareness: digital format suitability

### Adapting to the Digital Working Environment

#### Overview

An increasingly digital working environment has enabled the HC to continue their everyday work even during ordeals such as the COVID-19 pandemic. However, it was stated that as an HCP, there was a need to adjust to emerging organizational technological frameworks. In addition, support when facing technical issues was thought to be sufficient for some HCPs but insufficient for others. The importance of providing guidance and assistance during the digital workday was emphasized.

#### Cogwheels in the Digitalization Machinery: Importance of Support, Knowledge, and Preparations

Having to learn and understand digital tools and facilities and constantly being required to have up-to-date knowledge were experienced as challenging by most HCPs. In addition, some HCPs expressed a lack of education on the use of digital tools. Most HCPs stated that manuals and guidelines on how to conduct digital consultations to create a therapeutic alliance with the patient were not available but were desired, as guidelines could ensure that essential elements in the communication process would not become lost, thus helping to ensure the quality of communication.

Technical support and advice were thought to be well functioning, as almost all HCPs were satisfied with phone support and stepwise guides for digital tool use. Some HCPs experienced support from “super users” (colleagues with cutting-edge expertise) as very useful. However, hands-on guidance regarding technical issues was expressed as inadequate by few HCPs.

Regarding the adequacy of digital communication, most HCPs expressed the need to establish certain prerequisites for digital use within the organization; employees’ digital competence and capabilities (pertaining to digital methods and tools) need to be sufficient to function optimally. Adequate patient digital literacy, that is, competence from the initial log-in step; the ability to manage digital tools well; and digital capabilities such as having a stable internet connection with adequate video and audio quality and up-to-date software and digital devices were understood by most HCPs to be vital to their work.

#### Workplace-Accelerated Digitalization: Opportunities and Challenges for the Professional Role

Most HCPs expressed that when meetings with colleagues were held in a digital-physical setting (blending digital participation and physical participation), it led to less interaction between attendees, occasionally leaving some attendees feeling that they had not been part of the meeting. When attending digital conferences, it was expressed that it was difficult to visually raise hands and share thoughts, partly because of a fear of “social clumsiness,” which resulted in feelings of frustration. Conversational turn-taking was experienced as time consuming, yet it was considered necessary in some groups.

It was perceived that digitalization has developed into a natural part of how health care could (and maybe should) be delivered. Some HCPs experienced gratification in having been quickly “thrown into” an increasingly digitally managed organization because of the COVID-19 pandemic, as this enabled them to assess how digital workdays might be arranged in the future. However, some HCPs observed a certain degree of skepticism toward digitalization as part of quality management among colleagues, but the acceptance of digitalization seemed to be dominant.

Most HCPs experienced increased cognitive fatigue and tiredness because of the increasing number of digitally held conferences and consultations. The need to learn new technologies and working routines was constantly ongoing, which could also contribute to cognitive fatigue. Technical errors were a source of frustration and stress, and some HCPs mentioned that technical errors caused a sense of hopelessness.

Some HCPs experienced working from home as positive, as this type of setting minimized stress and eased everyday life coordination, for instance owing to time savings. The increasing number of digital consultations at the HC allowed a considerable portion of planned activities to be maintained, thus enforcing accessibility despite the COVID-19 pandemic, and most HCPs stated that the digital working day was desirable as a way to continue working in the future. However, the digital working day was considered a suitable complement to more traditional ways of working that relied heavily on physical presence, as the more traditional way of approaching health care was stated as important because of the varying levels of digital literacy among patients and providers.

### The Difference Between Replacement and Complement

#### Overview

The HCPs found digital consultation to be a suitable complement in many cases and settings. However, consideration of the suitability of the digital format to the specifics of each particular case was expressed as crucial. The importance of having additional, more traditional ways of contacting HCPs was emphasized.

#### Emphasizing the Flexibility of Digital Communication: The Digital Format as a Facilitator

Most HCPs pointed out that individuals diagnosed with neuropsychiatric disorders might benefit from communicating digitally with HCP, as attending digital consultations from their home environment might be comforting, enabling a dedramatizing, informal setting. Digital consultations were described as most suitable for individuals with anxiety or other social, psychiatric, or mental health impairments owing to the flexibility and accessibility features of the digital format. Digital consultations were considered to not be as mentally exhausting and intense for the patient as face-to-face consultations.

It was expressed that a digital consultation for an initial patient contact could promote future face-to-face consultations at the clinic, as digital communication was thought to encourage the establishment of the HCP-patient alliance in some cases. In addition, some patients were considered to be more open when they were met through a digital format, which was mainly acknowledged when patients were located in a home environment while they were attending the consultations. Attending from familiar environments was perceived as giving patients a sense of safety. This was thought to be especially applicable to individuals diagnosed with neuropsychiatric disorders. Some HCPs experienced digital consultations as reducing both the rescheduling and cancelation of appointments.

When arranging digital conferences, some HCPs found it easier for different parties to agree on a consensual suitable time for digitally held meetings than for meetings held in a physical setting. A few HCPs mentioned that the nervousness that they sometimes experienced before meetings with colleagues was reduced, as digital conferences reduced the feeling of being “reviewed” by other meeting attendees. It was considered easy and thus positive to be able to connect to various meetings digitally (with parties such as patients and stakeholders), also acknowledging the environmental and time-saving benefits associated with decreased traveling. The digital format for meetings was considered to enable a more efficiently planned workday.

#### Having a Case-by-Case Awareness: Digital Format Suitability

Almost all HCPs expressed concerns about individuals with insufficient digital literacy, as this was thought to be related to health care accessibility. Some concerns were raised about individuals with poorer health, as a few HCPs reported that poor health might impair cognitive ability, which in turn could negatively influence the patient’s capability of using digital tools. The importance of additional means of contacting HCPs in addition to formats was thus stressed, as health care accessibility was considered crucial in affecting individuals with insufficient digital literacy.

Digital consultations occasionally caused some HCPs to question and worry about their ability to make accurate assessments digitally, as it was difficult to acknowledge “vital signs” of importance to the clinical assessment, such as visual indications of abuse, or ill-health, such as pale skin and skin rashes. Ensuring patient safety was considered complicated because of the impaired ability to make a holistic evaluation of individuals based on bodily expressions that may indicate restlessness and nervousness during digital consultations with patients. In addition, most HCPs stated that the digital format impaired interpersonal interaction, impacting nonverbal cues such as bodily expressions and gestures, as these were diminished to some extent. Important elements such as being able to better observe or better understand individuals’ interaction and communication with their surroundings were considerably affected and were considered by some HCPs to have a potentially negative effect on the HCP-patient alliance.

A few HCPs expressed concerns about the suitability of prescribing medication based on digitally conducted assessments and also raised concerns about being unable to conduct medication follow-ups, as the digital format made it difficult to make before and after comparisons of patient restlessness or other factors that may be affected by medication, because of the diminished availability of nonverbal cues.

Some HCPs experienced that some forms of consultations required a physical meeting space (such as the HC) to create a safe and comfortable environment for patient communication. Nearly all HCPs emphasized the need to assess whether a digital consultation was appropriate for each case, stating that the digital format is not suitable for everyone or suited to everyone or for every purpose. For example, digital consultations were considered to be unsuitable for patients who easily become unfocused or exhausted during the meeting. According to all HCPs, the inability to illustrate while interacting with patients and the use of whiteboards as examples of communication-promoting tools were experienced as negatively affecting communication quality. Almost all HCPs felt that digital consultations did not enable detailed communication or promote in-depth dialogue to the same extent as in a physically held consultation. Some HCPs expressed that this was affected by the reduced dynamic between meeting attendees and the fact that, although preferred by some, others need physically held consultations to be able to open up in more when communicating.

A few HCPs experienced that digitalization should not only be considered for individuals who are already skilled; even digital novices might benefit from the digital format if they learned to take advantage of it, in accordance with sufficient digital literacy. Putting the patient’s perspective first in managing their care was considered central to this issue, as some HCPs also stated that health care employees need to be encouraging and offer help and support in this transformation toward an increasingly digitalized context. Furthermore, it was the view of HCPs that patients often needed assistance in their initial forays into digital tools and facilities, and supporting individuals in this way was sometimes considered time consuming for some HCPs, but still worth the effort, as promoting more digital skills in both patients and providers was perceived as enabling more efficient management of health care.

## Discussion

### Principal Findings

The main results of the study showed that maintaining activities within the HC owing to organizational development (prompted by the COVID-19 pandemic) required HCPs to be part of an increasingly digitally managed workplace. HCPs were constantly influenced by the overall theme of this study: having to balance between the digital and physical work activities during a workday and having to constantly review digital communication as a complement versus as a replacement. Thus, the digital format was not perceived as suitable for everyone, at every time, or for every purpose, although digital communication was often perceived as a useful complement in many cases and settings among the HCPs in this study.

The results of this study will be discussed from 2 viewpoints: the patient contact perspective and the workplace perspective, with an additional focus on collegial interactions.

### The Patient Contact Perspective

In this study, nearly all HCPs emphasized the importance of acknowledging the difference between replacement and complement regarding the use of digital consultations and of acknowledging its varying suitability. For example, individuals diagnosed with neuropsychiatric disorders were considered to benefit from the digital format when communicating with HCPs, as individuals diagnosed with neuropsychiatric disorders might feel more comfortable attending digital consultations from an already well-known environment as opposed to attending face-to-face consultations at the clinic, which might be less familiar. Digital consultations could also enable a dedramatizing, less stressful, and more comfortable meeting environment for patients compared with face-to-face consultations. In addition, previous research suggests that patients experience a chat function for health care communication purposes as more considerate of their conditions compared with face-to-face communication [25]. It has also been previously suggested that interaction with HCPs via a digital platform (including chat-based features and phone calls) might encourage patients to open up more easily and share their thoughts more owing to increased engagement from HCPs [26].

In contrast, some HCPs in this study found it difficult to foster in-depth communication during digital consultations. Previous research suggests that health care delivered digitally will affect the HCP-patient alliance in different ways [27], as the use of nonverbal cues such as bodily expressions and gestures stimulates the establishment of the HCP-patient alliance [28]. Nonverbal cues offer insight into the underlying unstated concerns and emotions and also support the reinforcement or contradiction of verbal communication [29]. In addition, most HCPs experienced that interpersonal interaction was diminished during digital consultations, as nonverbal cues were minimized. The negative effect of the digital format on these aspects of communication has been previously suggested [30], which implies that over time, these aspects could negatively affect treatment [31,32]. In contrast, it was previously suggested that telemedicine provides similar [33] or better outcomes than conventional face-to-face health care [34], but always assessing the suitability of a digital consultation in each individual case is thought to be of utmost importance [35]. The latter supports the findings of this study, as HCPs stressed the importance of always carefully considering the appropriateness of digital communication from the perspective of each patient.

Almost all HCPs experienced that there were no easily accessible guidelines provided by their organization about how to best conduct digital meetings. Previous research emphasizes the use of guidelines and understanding a patient’s previous digital experiences and digital literacy [36]. Similarly, the results of this study indicate that digital literacy and sufficient digital capabilities are required when conducting a digital consultation. Along with digital capability requirements, such as managing digital methods and tools [37], HCPs experienced a need to adopt the role of patient educators in digital matters. Previous research implies that it may be challenging for individual clinics to handle digital transformational processes, leaving HCPs to improvise and individually evaluate whether health care can be safely delivered [38]. In addition, previous research also suggests that, to prevent digital exclusion, HCPs should offer information, encouragement, or tools for patients [39], which is in accordance with the findings suggesting that tailored education for individuals is needed within the telehealth domain [40].

Most HCPs highlighted the lack of opportunities to complement verbal communication, such as drawings to assist verbal communication and the use of whiteboards. These communication-promoting tools were normally used at the HC and were considered to highlight and concretize verbal HCP-patient communication. Technologies are constantly undergoing development, modern videoconference tools provide virtual white boards as integrated parts of the software [41], and previous research suggests that virtual whiteboards support children’s collaborative communication abilities in a classroom setting with reference to the communication process and use of a “learning resource” as part of integrated system of spoken dialogue and nonverbal communication [42]. Hence, the use of digital whiteboards as part of an integrated videoconference tool might constitute a possible alternative to more traditional communication-promoting tools such as paper and pencil or physical whiteboards.

To tackle these challenges, guidance [43], support for developing and maintaining HCP-patient alliances [44], and methods of how to conduct best practices in the context of increasing digitalization need to be addressed [45]. Raising awareness of the risks of digitalization of health care is vital, as is adopting techniques for sustainable digital clinical relationships [44]. In addition, previous findings indicate that HCPs are generally positive about implementing therapeutic initiatives digitally. Although attitudes might have been influenced by previous experiences related to the clinic and changes within and previous digital format use, feelings of fatigue, incompetence, and insecurity were expressed, as were experiences of having reduced physical contact with the patient [46].

### The Workplace Collegial Perspective

Some HCPs experienced frustration with technological failures and having to rely on their own insufficient expertise, which conforms to previous findings that digitalization is not always implemented in ways that take digital competence into account. This is an important point to take seriously, as having insufficient technological competence might cause frustration among HCPs when trying to adopt new technologies [47]. In addition, the accelerated changes forced by the rapid introduction of digitalization were not experienced as being followed by sufficient professional training and guidance. This has been previously demonstrated within the telerehabilitation field [36] and might be important, as HCP competence and willingness are crucial factors for successful health care digitalization [48]. Furthermore, some of the recent rapid uptakes of digital tools in Swedish health care have been accepted, as this aid was prompted by exceptional circumstances. Considering these digital tools as possible benefits in health care seems to depend to a large extent on individual and organizational aspects, rather than on technological aspects alone [49]. However, HCP education regarding how to conduct digital consultations has not been extensively realized [14], although it has been previously suggested that HCPs need training regarding newly implemented tools [50], for example, in learning how to efficiently communicate digitally in clinical matters [47,51].

To harmonize digitalization within the health care sector, assessing and improving HCPs’ digital competencies might be an initial step in incorporating digitalization effectively into clinical practice. This is important from an organizational development perspective, as providing the best possible health care for patients requires HCPs to develop profound knowledge and skills in relation to new working ways prompted by digitalization [52]. Previous research also pinpoints that employee’s social relations might influence behavior and acceptance of novel technologies [53], which agrees with the findings of this study, seen in the positive experiences that some HCPs experienced regarding receiving help from colleagues with special competence, the so-called “superusers.”

Although situations such as the COVID-19 pandemic might stimulate the rather prompt and less prepared implementation of telehealth solutions within health care [54], the HCPs in this study experienced being hastily thrown into the digitally working climate as quite positive, having the advantage of being able to pilot future possible working methods. Although the rapidly emerging situation seemed to be slightly amorphous at first, the HCPs emphasized the flexibility of the digital format. This corresponds to previous findings indicating that quickly adopting digital solutions may catalyze telehealth development within organizations [38].

As part of an increasingly digital working environment, working from home was perceived as advantageous owing to the increased flexibility experienced by most HCPs. It was considered easy and thus positive to be able to connect to various meetings digitally, which was also expressed as positively related to the environmental benefit attributed to decreased business travel, which has been previously reported [55,56]. Most HCPs were relatively positive toward digitalization as a future feature of the workplace, embracing its presence and accepting digital solutions as potential answers to the ongoing and progressive health care management transformation. In the literature on digital tool assistance, the term “useworthy” emerged, aiming to demonstrate not only the usefulness of a technology but also to show its value as it meets the high priority needs of the users [57].

Digital conferences for workplace events such as staff meetings were experienced as convenient by most HCPs because of resource savings, scheduling advantages, and increased accessibility. In accordance with these findings, previous research suggests that telehealth technology use enables HCPs to more easily share information and collaborate in patients’ treatment [58], improving interdisciplinary collaboration [59], thus helping to overcome collaboration barriers [60] and ensure continuity of care.

Always being up-to-date regarding software and holding nearly all conferences and consultations digitally were perceived as tiring to some HCPs. Furthermore, our findings imply that this increased tiredness prevails in part because of an increasingly digitalized working environment, which has previously been explored in terms of workplace digital fatigue [61,62], more specifically mentioned as “videoconference fatigue” because of increased participation in digitally held meetings [63]. However, it is important to emphasize that for any meeting to be successful, in-person and digital meetings require adequate preparations, such as sharing relevant documents and agendas before the meeting [41]. Although videoconference fatigue conceptually belongs to the more common construct of “work fatigue,” the 2 concepts differ, as work fatigue is mainly associated with workplace demands in general, such as work overload and time constraints [64]. These features may also apply to videoconference fatigue, but as a concept, videoconference fatigue is more specific than the general causes of work fatigue and could conversely be generated as a consequence of single events. For example, being active digitally imposes avoidance of technology-based distractions, while also calling for greater attention to be paid owing to fewer available nonverbal communication cues [65]. This is in accordance with the findings of this study, implying that most HCPs experienced that cognitive fatigue, besides tiredness, occurred because of an increased number of digitally held conferences and consultations.

In this study, closely related to having to balance the digital-physical workday, digital solutions used within the organization correspond to the value of technology and thus its “worth,” as using technology fulfills the clinic’s needs, maintaining valuable HCP-patient contact while keeping the focus on patients. As a knowledge gap seems to exist regarding habilitation services overall, our purpose was to further explore the field of habilitation explicitly. We would also like to highlight that more research is needed within the domain of telehabilitation, both from an HCP and a patient perspective, with respect to the multifaceted profile of HC clients. Moreover, to apply a broader perspective, a macrolevel insight would be useful to obtain, for example, by inquiring into the managerial viewpoint of digitalization in (clinic-specific) health care further exploring the use of digital tools and digitization in health care in general from a managerial perspective, implementation strategies may be worth exploring. This may be particularly relevant considering the importance of sustainable health care management and development. Emphasizing core values in health care, that is, health care should undergo constant quality improvements [66], with a focus on aspects such as patient engagement, patient-centeredness, and health literacy [67], future research may focus on digital tool use in matters of clinical assessments, more explicitly regarding how best to conduct clinical assessments sufficiently via a digital format as a part of digital consultations. On the basis of the results of this study, we propose that future research may target whether digital formats mediate a holistic view of an individual and adequately provide HCPs with sufficient amounts of clinical information to take further actions, for instance, regarding drug prescriptions or further referrals in health care. This question is of great significance to patient safety, as digital health care consultations might be less appropriate for some patient conditions or when technological shortcomings suddenly interrupt consultations [68]. The issue of patient safety is also very important for HCPs, whose professional role includes showing empathy and compassion as well as professional integrity [69].

### Methodological Considerations

In this study, the HC’s operations developer was contacted and asked to arrange initial contact with potential participants. Using the operations developer at the HC for participant recruitment could be associated with an ethical risk [70], for instance, as some participants may have felt prompted to participate, thereby not fully conforming to the rule of voluntary participation. There is an additional risk of biased sampling, as the person assisting in the recruitment process may easily become too helpful, wanting to recruit participants who they think can provide suitable answers [70]. However, participants were not recruited directly by the operations developer; they were asked to contact 1 of the researchers via the operations developer if they were interested in participating after being given additional information about the study at the web-based workplace meeting. Furthermore, self-selection bias may also occur because of personal engagement in the digitalization process at the HC, in accordance with participants’ specific characteristics; thus, some HCPs might have been more likely to take part in the study than others. However, self-selection bias is difficult to avoid completely in interview-based research because voluntary participation is central to ethical good practices [71]. Furthermore, people who volunteer in this way are more likely to have things on their mind, positive aspects as well as negative, that they would like to convey; hence, they are probably better informants in an interview study.

All data were collected using videoconferencing. Previous research supports the use of digital methods for qualitative data collection because of their cost-effectiveness, security options, ease of use [72], and relaxed environment, which can occasionally foster deepened conversations [73]. In contrast, feelings of videoconference fatigue may have potentially affected participants’ willingness to participate in a digital interview [63]. Moreover, interviews conducted in a digital form might exclude some populations owing to varying levels of digital literacy [72], and the digital format also limits researchers’ opportunities to fully observe the full range of body language and nonverbal communication [74]. Selective transcription was used in the analysis process, as suggested by Halcomb and Davidson [19], following their guidance on the analysis process. Using audio recordings along with memo writing might help assure methodological accuracy in terms of credibility [75], as memo writing was complemented by listening to recordings when conducting the 2-step content analysis [19]. The use of written memos conducted either during an interview or immediately afterward has been suggested to be superior to only the use of audio recordings transcribed verbatim in terms of enhanced trustworthiness [76]. The fact that the 2 researchers who conducted the interviews also reached a consensus during the analytical phase further contributed to this study’s trustworthiness [77]. It can be concluded that data saturation [78] was reached when looking through and reflectively discussing the notes shortly after the last 3 interviews. At this point, the researchers estimated that no further information would be obtained [79]. To have most of the study population defined by only 1 gender might, however, affect the point at which data saturation was reached. However, it has previously been shown that studies with relatively homogenous study populations may reach reliable saturation at quantities similar to those in this study [80].

### Conclusions

Managing a workday influenced by the balance between digital and physical demands forces HCPs to adjust to the digital format as part of an increasingly digitally managed workday. Being aware of digitalization as a workplace development process and constantly having to adapt to changing demands (considering that digital formats are not suitable for every patient encounter) is a complex yet required task. Driven by professional values such as putting patient care first, negotiating the pros and cons of health care digitalization is a constantly evolving and challenging process.

Therefore, having to balance work that bridges both the physical and digital work activities in times of rapid organizational development fueled by digitalization spurs the need to acquire knowledge on the adoption of new ways of working. In an HC setting, the introduction of digital tools to increase knowledge and the possibility of tailoring patient visits to different patient populations, such as individuals diagnosed with neuropsychiatric disorders, is likely to complement more traditional ways of practicing medicine.

## Abbreviations

COREQ: Consolidated Criteria for Reporting Qualitative Studies
HC: habilitation clinic
HCP: health care professional
